# Genetic Vaccination as a Flexible Tool to Overcome the Immunological Complexity of Invasive Fungal Infections

**DOI:** 10.3389/fmicb.2021.789774

**Published:** 2021-12-15

**Authors:** Laura Luberto, Bruna Neroni, Orietta Gandini, Ersilia Vita Fiscarelli, Giovanni Salvatori, Giuseppe Roscilli, Emanuele Marra

**Affiliations:** ^1^Takis s.r.l., Rome, Italy; ^2^Cystic Fibrosis Diagnostic Section, U.O. Microbiology and Immunology Diagnostic, Department of Immunology and Laboratory Medicine, Children’s Hospital Bambino Gesù Organization IRCCS, Rome, Italy; ^3^Department of Molecular Medicine, Sapienza University of Rome, Rome, Italy

**Keywords:** genetic vaccination, invasive fungal infections, aspergillosis, vaccine, immunotherapy, anti-fungal, SOT, HSCT

## Abstract

The COVID-19 pandemic has highlighted genetic vaccination as a powerful and cost-effective tool to counteract infectious diseases. Invasive fungal infections (IFI) remain a major challenge among immune compromised patients, particularly those undergoing allogeneic hematopoietic bone marrow transplantation (HSCT) or solid organ transplant (SOT) both presenting high morbidity and mortality rates. Candidiasis and Aspergillosis are the major fungal infections among these patients and the failure of current antifungal therapies call for new therapeutic aids. Vaccination represents a valid alternative, and proof of concept of the efficacy of this approach has been provided at clinical level. This review will analyze current understanding of antifungal immunology, with a particular focus on genetic vaccination as a suitable strategy to counteract these diseases.

## Introduction

Nearly a billion people are estimated to have skin, nail, and hair fungal infections and more than 150 million people have serious fungal diseases, which have a major impact on their lives or are fatal. Aspergillus and Candida remain the main fungal pathogens responsible for a high number of cases of serious fungal disease (Schmiedel and Zimmerli, 2016). Mortality for these types of infection range between 25 and 90%, depending on the severity of the underlying clinical conditions (Lass-Flörl, 2009). The major target of invasive aspergillosis and candidemia are patients who experience hematopoietic bone marrow transplantation (HSCT) and Solid organ transplant (SOT). Chronic pulmonary aspergillosis is globally estimated in 3,000,000 cases, ∼700,000 cases of invasive candidiasis, and ∼250,000 cases of invasive aspergillosis occur annually (Bongomin et al., 2017). The overall incidence of IFIs in hematopoietic stem cell transplant (HSCT) patients was 3.4%. Invasive aspergillosis (IA) is the most common IFI encountered in the HSCT population: Aspergillus accounted for 43% of infections and Candida accounted for 28%. The remaining 29% is associated to other fungal infections (Kontoyiannis et al., 2010). In allogeneic HSCT recipients, there are three periods of risk for IA: (1) neutropenia after the conditioning regimen; (2) exogenous immunosuppression for prevention or treatment of acute Graft Versus Host Disease (GVHD); and (3) exogenous immunosuppression for treatment of chronic GVHD (after day 100 following transplant) (Person et al., 2010). Among solid organ transplant (SOT) recipients, more than 50% of all fungal infections were due to Candida, except for lung transplant recipients, where Aspergillus was the most common fungal pathogen, and which were responsible for 70% of IFIs (Marr et al., 2002). Unquestionably in SOT patients, rejection and exogenous immunosuppressive agents, particularly high-dose steroids and antilymphocyte treatments, lead to an increased risk of the onset of IFIs in this population (Gavaldà et al., 2014). Despite current antifungal therapies there is still a considerable proportion of cases of fungal infections that remain without a cure. Antifungal resistance represents a major clinical challenge for treating invasive fungal infections (IFI) that could be as high as 20 (Wiederhold, 2017). Current antifungal drugs may be limited by drug–drug interactions and serious adverse effects/toxicities in severely ill patients and prevent their prolonged use or dosage increase (Robbins et al., 2016; Tverdek et al., 2016). Thus, enlarging the arsenal of systemically available antifungals and developing alternative strategies to pharmacological intervention becomes increasingly necessary. Immunotherapies represent a valid alternative or a synergic support to current antifungal (Robbins et al., 2016; Tverdek et al., 2016) therapies and include a wide range of approaches (Sam et al., 2018; Table 1). Despite preclinical evidence of the efficacy of vaccination in both bone marrow transplanted and corticosteroid immunosuppressed mice (Ito and Lyons, 2002; Bozza et al., 2009), the main issue to be solved is whether vaccination could be an effective strategy to prevent and cure fungal infection also in patients whose immune system is severely impaired/compromised (Scriven et al., 2017).

**TABLE 1:** Summary of the attempted immunotherapies to date.

Immunotherapeutic approaches in fungal diseases			

Strategy	Target pathogen	Experimental study level	References
**Recombinant cytokines and immune activating compounds**			
Colony-stimulating factors (G-CSF, GM-CSF)	*Candida*, *Cryptococcus*	Preclinical and clinical	Safdar et al., 2013; Mezidi et al., 2014; Wan et al., 2015
IFNγ	*Aspergillus, Candida*	Clinical	Nagai et al., 1995; Sainz et al., 2007; Buddingh et al., 2015
TNF-α	*Aspergillus*	Preclinical and clinical	Romani et al., 1997; Mehrad et al., 1999
IL-12	*Candida albicans, Cryptococcus neoformans*, *Aspergillus fumigatus*	Preclinical	Decken et al., 1998
IL-15	*Candida albicans*, *Aspergillus fumigatus*, *Fusarium* spp., and *Scedosporium* spp.	*In vitro*	Musso et al., 1998; Winn et al., 2003
Thymosin-α-1 (Tα1)	*Candida albicans*	Preclinical	di Francesco et al., 1994
**Cell therapy**			
Neutrophil enrichment by Granulocyte Transfusion (GTX)		Clinical	Seidel et al., 2008
Dendritic cell (DC) immune therapy	*Aspergillus fumigatus*	*In vitro* and preclinical	Banchereau and Steinman, 1998; Ramirez-Ortiz et al., 2011
NK cell therapy	*Aspergillus fumigatus, Candida albicans*	*In vitro* and clinical	Voigt et al., 2014; Stuehler et al., 2015
Adoptive T cell transfer	*Aspergillus*	Clinical	Perruccio et al., 2005; Bacher et al., 2015
Chimeric antigen receptor (CAR) T cell therapy	*Aspergillus*	Preclinical	Kumaresan et al., 2014
**Antibody therapy**			
Anti-beta-glucan monoclonal antibodies	*Candida albicans*	*In vitro* and preclinical	Torosantucci et al., 2009
Anticryptococcal monoclonal antibody	*Cryptococcus neoformans*	Preclinical and clinical	Larsen et al., 2005; Rachini et al., 2007
**Checkpoint inhibitors**			
	*Aspergillus*	*In vitro* and preclinical	Stephen-Victor et al., 2017
**Vaccination strategies**			
**Live attenuated virus**			
Formalin killed *Coccidioides immitis* spherules	*Coccidioides immitis*	Preclinical and clinical	Ardiani et al., 2010
Heat killed *Saccaromyces cerevisiae* (KHS)	*Coccidioides posadasii, Candida albicans, Aspergillus fumigatus*	Preclinical	Capilla et al., 2009; Ardiani et al., 2010; Liu et al., 2011
Attenuated strain of *Coccidiodes posadasii*	*Coccidioides posadasii*	Preclinical	Xue et al., 2009
Deletion of *Blastomyces adhesion-1* (Bad-1) gene	*Blastomyces dermatitidis*	Preclinical	Brandhorst et al., 1999; Wüthrich et al., 2000
Attenuated strain of *Criptococcus neoformans* (H99g)	*Cryptococcus neoformans*	Preclinical	Cox and Magee, 1998; Wozniak et al., 2011
Live attenuated strain of *Tricoptyton verrucosum*	*Trichophyton verrucosum*	Preclinical	Gudding and Naess, 1986
**Conjugates**			
B-glucan polysaccharide from brown algae with inactivated diphtheria toxin (CRM)	*Candida albicans*, *Aspergillus fumigatus*	Preclinical	Torosantucci et al., 2005
B-1,2 mannotrios with fructose biphosphate aldolase (Fba)	*Candida albicans*	Preclinical	Xin et al., 2012
Capsular polysaccharide antigen of *Cryptococcus neoformans* (GXM) with tetanus toxoid	*Cryptococcus neoformans*	Preclinical	Devi, 1996
**Recombinant/subunit**			
NDV-3 Agglutinin-like sequences (Als3-p) for *Candida* spp.	*Candida albicans*	Clinical	Phan et al., 2007
Secreted aspartyl proteinase-2 (Sap-2) for *Candida* spp.	*Candida* spp.	Preclinical and clinical	De Bernardis et al., 2012; Santos and Levitz, 2014

## Vaccination Against Fungal Infection

Our understanding of fungal immunity have increased the prospect of developing vaccines against fungi that are effective, safe, and able to elicit lasting immunity even in immune deficient individuals (Espinosa and Rivera, 2016; Nami et al., 2019). Genetic immunization has been shown to induce humoral as well as cellular immune responses with high efficacy (Bolhassani and Yazdi, 2009) and has the potential to guide the host immune response by co-expressing immunomodulatory and costimulatory molecules (Kutzler and Weiner, 2008). Genetic vaccines have been widely used against numerous pathogens, and current experience with COVID-19 has raised new hopes about the effective use of this strategy in other infectious diseases (Abu Abed, 2021). Genetic vaccination offers the opportunity to easily screen for a wide number of microbial antigens, thus enhancing the chances of success. Considering the complexity of the host immune response to the fungus, particularly in immunocompromised patients, a highly flexible vaccination strategy is extremely desirable. The sequencing of fungal genomes has identified key functional factors essential to fungal survival and virulence in the human host (Iyalla, 2017; Priest et al., 2020) and has helped to understand how the fungus evades the immune system or establishes infection (Hogan et al., 1996). This analysis would be of help in at least four different ways. First, an ideal target would protect against infection from multiple species of fungi by containing conserved epitopes that elicit both T and B cell responses. Second, sequence comparison of genes from different fungi may help to identify cluster-related genes with the potential of becoming the targets of pan fungal vaccines with a broad spectrum of activity. Studies on preclinical models of pan vaccination have demonstrated a protective cross reactive immune response against different fungi (Hamad, 2012; Champer et al., 2016). Third, sequence analysis would help to identify the best immunogenic proteins, preferably those secreted or expressed on the cell wall (Musso et al., 1998). Fourth, given the possible homologies between fungal and human proteins, the fungal target sequence selected must be markedly dissimilar to any human one (Edwards, 2012). A proof of concept that vaccination is an effective strategy to counteract fungal diseases is provided by studies in several models of invasive fungal infection. Vaccination with recombinant Aspergillus fumigatus Asp f3 (Pmp20) protected mice from aspergillosis following neutropenia or corticosteroid induced immunosuppression (Bozza et al., 2002; Ito et al., 2006, 2009; Diaz-Arevalo et al., 2011, 2012). Vaccine formulations with Crf1 Gel1, and Pep2 (Bozza et al., 2009; Stuehler et al., 2011) provided protection against aspergillosis in comparable experiments. Protection from candidemia has been conferred by immunization with recombinant Mdh1, Sap2, and Als3 (Vilanova et al., 2004; Spellberg et al., 2008; Shibasaki et al., 2014) with the last two investigated in clinical trials (Schmidt et al., 2012; Cassone, 2013). However, the use of recombinant fungal proteins for vaccination has some important limitations which in part explain why no anti-fungal vaccine has been approved to date by a regulatory agency. Processes to produce a recombinant protein could be cumbersome, requiring costly resources and lengthy production times. In this context genetic vaccination could represent a valid strategy for antifungal vaccination.

### Genetic Vaccination—General Features

Cross talk between innate and adaptive immunity arms of the immune system is essential for the resolution of fungal infection. Interruption of this connection by iatrogenic drugs or depletion of key immunity cells due to ablative chemotherapies imbalances the host immune response and predisposes to fungal infection. In IFI, Th1-biased based immune responses correlate with protective immunity and resistance, whereas type 2 helper T (Th2)-based responses generally lead to an exacerbation of the disease. Th1 cell activation is instrumental to clearing infection by improving the effector function of innate immune cells through the release of pro-inflammatory cytokines (Pathakumari et al., 2020). Any vaccination strategy must be able to redirect this imbalance toward an effective immune response against the fungus. Genetic vaccines have become an attractive approach for generating antigen-specific immune responses (Bolhassani and Yazdi, 2009; Flingai et al., 2013). A protein expressed by a genetic vaccine displays its native conformation with the relevant post-translational modifications, which are required to elicit both humoral and cell mediated neutralizing immune responses to conformational epitopes (Bolhassani and Yazdi, 2009). Genetic vaccines can be easily prepared on a large scale with high purity and stability in relation to proteins and other biological polymers. In addition this strategy allows artificial immunogens and co-expression of immunomodulatory proteins to be engineered (Sharma and Khuller, 2001). Among these: targeting antigens for rapid intracellular degradation, directing antigens to APCs by fusion to ligands for APC receptors, co-translating antigens with chemokines and cytokines or with co-stimulatory molecules and coadministration with CpG oligonucleotides (Sharma and Khuller, 2001). The plasticity of genetic vaccination could be of importance in those contexts where immunosuppressive therapies alter the normal homeostasis of the innate and adaptive immune system. This type of approach has been evaluated in other fungal infections such as Paracoccidiodomycosis, Coccidiomycosis, and Pneumocystosis (Ivey et al., 2003; Zheng et al., 2005; de Amorim et al., 2013).

### Genetic Vaccination on Fungal Infection

#### Paracoccidioidomycosis

Paracoccidioidomycosis (PCM) is an important endemic mycosis in Latin America with significant morbidity and mortality (Marques et al., 2006; de Almeida et al., 2018). There are two recognized *Paracoccidioides* species, *P. brasiliensis* and *P. lutzii*. Approximately 1,853 (∼51.2%) of 3,583 confirmed deaths in Brazil due to systemic mycoses from 1996 to 2006 were caused by PCM. Antifungal treatment is required for patients with PCM. The initial treatment lasts from 2 to 6 months and sulfa derivatives, amphotericin B, azoles and terbinafine are used in clinical practice; however, despite prolonged therapy, relapses are still a problem. An effective Th1-biased cellular immune response is essential to control the disease, which can be induced by exogenous antigens or modulated by prophylactic or therapeutic vaccines (Taborda et al., 2015). An initial vaccine consisting of 15 amino acid peptides, named P10 peptide, derived from the immunodominant antigen gp43 of *Paracoccidioides brasiliensis* was evaluated in a mouse model of intratracheal infection. This peptide was shown to elicit a protective Th-1 response and, when used in combination with the standard chemotherapy regimens for experimental PCM, improved treatment efficacy (Taborda et al., 1998; Marques et al., 2006; Silva et al., 2008). Successively, a plasmid DNA containing the minigene encoding the P10 peptide, which includes the T-cell epitope of gp43, was evaluated in the same model of infection. Animals immunized with the plasmid pcDNA3-P10 showed a significant reduction in the pulmonary fungal burden when compared to non-immunized and only plasmid vector immunized mice after intratracheal infection. This vaccine increased the percentage of CD4^+^ and CD44hi memory T cells and Foxp3^+^ Treg cells in the spleens and lungs of immunized mice, reinforcing the concept that the presence of T regulatory cells upon secondary antigen exposure may prevent immunopathology in the context of vaccination and favor long-term memory. Furthermore, the pattern of cytokines released by the splenocytes from mice immunized with pcDNA3-P10 is consistent with a Th1-biased T-cell immune response (de Amorim et al., 2013), which is predictive of a positive clinical response as discussed above.

#### Coccidioidomycosis

*Coccidioides immitis* and *C. posadasii* are two highly pathogenic dimorphic fungal species that cause coccidioidomycosis (also known as Valley Fever). They are endemic in the arid region from west Texas to southern and central California in the United States and up to 50% of long-term residents get infected. Pulmonary symptoms are the most common, but it is estimated that only 30–50% of infections are symptomatic. Fewer than 5% of immunocompetent patients develop disseminate disease. *Coccidioides immitis* or *Coccidioides pusadassi* are prime candidates for vaccine development (Yoon and Clemons, 2013). Primary infection, acquired *via* inhalation of the arthroconidia, is manifested by a benign or asymptomatic infection; however, others can go on to develop acute or chronic disease involving the lungs and/or extrapulmonary organs (Galgiani et al., 2005; McCotter et al., 2019). The acquired immunity that develops after active infection testifies to the feasibility of a vaccine for this disease (Cox and Magee, 1998). In 1995, Barry et al. (1995) published a technique, termed “expression library immunization” (ELI) for identifying protective genes. Immunizing with sequentially divided protective fractions of a genomic library has the potential to screen every gene in the pathogen’s genome and to offer the advantage of presenting the host with multiple genes, thereby simulating the effects of a live organism. Using ELI, investigators have identified protective genes in in a number of infection diseases (Barry et al., 2004). Following this type of approach, Ivey et al. (2003) identified a Coccidioides gene named ELI-Ag1 that has protective capacity in BALB/c mice against intraperitoneal challenge with 2,500 arthroconidia of this fungus. The gene was isolated from an original pool of 800–1,000 genes by successive fractionation of a cDNA library prepared from parasitic spherule-phase cells. The ELI has proven to be a useful strategy for identifying protective gene pools and offers the decided advantage of not requiring *a priori* knowledge of whether protection is mediated by cell-mediated or humoral immunity.

Direct comparison of vaccine efficacy against Coccidioidomycosis between recombinant antigen 2 (Ag2), a *Coccidioides immitis* isolated glycoprotein, and the same antigen cDNA expressed in a plasmid vector was performed by Jiang et al. (1999a). Genetic immunization with the plasmid vector encoding Ag2 enhanced survival of BALB/c mice challenge with *C. immitis* arthroconidia while it did not occurred in mice immunized with the recombinant antigen. This genetic vaccine co-expressing IL12 and Ag2 enhanced protective immunity against *Coccidioides immitis* through the induction of Th1-associated immune responses (Jiang et al., 1999b).

#### Pneumocystosis

*P. jirovecii* is the etiological agent of Pneumocystis pneumonia (PCP), causing an asymptomatic or mild infection in the normal host but fulminate pneumonia in the immunocompromised host. It accounts for an estimated 10,000 hospitalizations in the United States and > 400,000 cases worldwide each year. Even with treatment, mortality rates approach 10–20%. An overview of the immune response to Pneumocystis and current progress on novel vaccines and therapies is discussed in Gingerich et al. (2021). Despite current strategies to treat HIV infection and its complications, Pneumocystis (PC) pneumonia remains a common clinical problem (Morris et al., 2004) thus there is a need to develop CD4^+^ T cell–independent therapeutic strategies to prevent this infection. It has been demonstrated that overexpression of IFN-γ can result in eradication of PC in the absence of CD4^+^ T cell help (Kolls et al., 1999), in part through augmenting IFN-g–secreting type I (Tc1) CD8^+^ T cell response (McAllister et al., 2004). Another molecule expressed on activated CD4^+^ T cells that is critically important for co-stimulation and CD4^+^ T cell help is CD40L, that activates DCs to influence CD8^+^ cytotoxic T cell (Banchereau and Steinman, 1998; Grewal and Flavell, 1998) and B cell (Clark and Ledbetter, 1994) immune responses. It has been demonstrated that CD40L gene modified DCs pulsed with *Pseudomonas aeruginosa* (PA) could stimulate naive B cells to produce anti-PA antibodies and confer protection against PA challenge. Furthermore, CD40L-modified DCs pulsed with PC resulted in a protective antibody response in CD4-depleted mice and protected them against a PC challenge (Keely et al., 2003), thus indicating that immunotherapeutic approaches may represent a valid alternative to counteract PC infection. Zheng et al. (2005) identified on the surface of PC a protein named Kexin and have used it to validate DNA vaccination in CD4-depleted mice. Immunization with plasmid expressing Kexin under CMV promoter resulted in significant anti-PC IgG1 and IgG2a titers in CD4-competent mice, whereas titers were significantly lower in CD4-depleted mice. In comparison, CD4-depleted mice immunized with a plasmid expressing both Kexin and CD40L demonstrated significantly higher titers of anti-PC IgG1 compared with CD4-depleted mice immunized with the plasmid expressing only Kexin. Mice immunized with a plasmid expressing only CD40L or an empty vector alone, demonstrated no detectable anti-PC titer. DNA immunization with Kexin/CD40L resulted in antibodies capable to protect in primary challenge experiments as well as in adoptive transfer experiments, and mediate opsonic phagocytosis of PC, which may be critical for its therapeutic effect. Furthermore, mice immunized with pKexin/CD40L showed significantly lower fungal burdens, with nearly 3-log greater protection compared to control CD4-depleted mice (Keely et al., 2003). These data provide clear proof of concept that DNA vaccination exploiting the expression of a key costimulatory molecule can support a host immune response also in the context of a CD4 deficiency.

## Conclusion

Several reasons underlie the paucity of vaccination approaches to counteract fungal infection in immunocompromised hosts and identifying a niche patient population who could benefit from a cost-effective vaccine strategy is problematic, thus discouraging any efforts in this direction. Because responses to fungi depend on both arms of the immune response, there are formidable obstacles to identifying what are the key elements in translating preclinical models to a successful human vaccine. The timing of vaccination must be tailored to the time frame of risk to developing the disease and to the immunological features of patients. Pre- and post-transplant vaccination are both possible in SOT patients, while post-transplantation is the only option in HSCT. In addition, the high costs of preparing recombinant antigens for use in human studies that meet standards for Good Manufacturing Processes, poses a relevant issue of cost and timing to registration. Genetic vaccination offers a high degree of flexibility that potentially addresses these issues.

Genetic vaccination allows for the rapid and simultaneous screening of suitable antigens, selecting a candidate target that potentially provides cross reaction with other fungal antigens, thus laying the basis for pan vaccine development. Recommendation for immunization of SOT patients, already in place for other infectious diseases (Blanchard-Rohner et al., 2019), would certainly benefit pre- post- transplant vaccination able to cover a wider range of fungal infections. Genomic vaccination offers the flexibility to potentially overcome the complexity of the host immune status, particularly evident in HSCT and SOT patients. Both SOT and HSCT share the polarization of CD4^+^ cells toward the Th2 phenotype that significantly correlates with the risk of contracting fungal infection (Elenkov, 2004). The most important CD4^+^ T cells in the antifungal immune response are the Th1 and Th17 helper T cells. Th1 helper T cells secrete the cytokines IFN-γ and TNF-α which not only are able to activate T and B lymphocytes, but also innate immune cells, such as neutrophils, macrophages, DCs, and inflammatory monocytes, to fight against invading fungi. Genetic vaccination has the potential to redirect CD4^+^ lymphocyte immune response to Candida and Aspergillus toward a Th1 phenotype, for instance by co-expressing with the selected antigen a Th1 polarizing cytokine such as γ-IFN whose beneficial effect in fungal infections has already been demonstrated (Nagai et al., 1995; Sainz et al., 2007; Buddingh et al., 2015). As seen with pKexin/CD40 vaccination against PC, genetic vaccination could be programmed to overcome the low CD4^+^ lymphocyte cell count by co-expressing with the fungal antigens’ costimulatory molecule able to vicariate CD4^+^ lymphocytes and trigger a CD8 T lymphocytes immune response. Recent experience with the pandemic virus SARS-CoV2 have shown that genetic vaccines speed up development times and abate costs (Shin et al., 2020). Both these features are of considerable importance in developing therapies that require continuous checks in the clinical setting to adapt to the complexity of the interactions between host and fungi.

## Author Contributions

GS and LL wrote the review manuscript. EM revised the manuscript. All authors read and approved the submitted version.

## Conflict of Interest

LL, GS, GR, and EM were employed by Takis s.r.l., that is a commercial entity. The remaining authors declare that the research was conducted in the absence of any commercial or financial relationships that could be construed as a potential conflict of interest.

## Publisher’s Note

All claims expressed in this article are solely those of the authors and do not necessarily represent those of their affiliated organizations, or those of the publisher, the editors and the reviewers. Any product that may be evaluated in this article, or claim that may be made by its manufacturer, is not guaranteed or endorsed by the publisher.
